# Extracorporeal Rewarming From Accidental Hypothermia of Patient With Suspected Trauma

**DOI:** 10.1097/MD.0000000000001086

**Published:** 2015-07-13

**Authors:** Tomasz Darocha, Sylweriusz Kosiński, Anna Jarosz, Rafal Drwila

**Affiliations:** From the Department of Anesthesiology and Intensive Care, John Paul II Hospital, Collegium Medicum, Jagiellonian University, Cracow, Poland (TD, AJ, RD) and Department of Anesthesiology and Intensive Care, Pulmonary Hospital, Zakopane, Poland (SK).

## Abstract

The use of extracorporeal membrane oxygenation is a new approach to rewarming patients with severe hypothermia and hemodynamic instability. There are, however, many questions regarding qualification for this technique in case of suspected or confirmed trauma.

A male with confirmed accidental hypothermia (25°C) and after successful cardiopulmonary resuscitation from in-hospital cardiac arrest was subjected to a protocol of extracorporeal rewarming from profound hypothermia. Because of unclear history, a full trauma computed tomography was performed that showed pericerebral hematoma and signs of previously undergone right craniotomy, multiple right-sided rib fractures and the presence of intraperitoneal fluid. Based on repeated imaging and specialist consultation, no life-threatening injuries were identified and rewarming with extracorporeal membrane oxygenation was safely performed. In a year follow-up, the patient was found to be alive, with no neurologic deficits.

Although this case highlights the first successful utilization of extracorporeal rewarming in a trauma patient at our center there are several limitations to its widespread use

## INTRODUCTION

Accidental hypothermia is a serious medical condition, which may be deleterious if not treated properly. Severe hypothermia with core temperature below 28°C and hemodynamic instability is frequently fatal, if standard rewarming techniques are to be used.^1^ There are, however, numerous case studies showing that the use of extracorporeal membrane oxygenation (ECMO) or cardiopulmonary bypass (CPB) enables to restore normal temperature, and, surprisingly, the neurological outcome, even with prolonged cardiopulmonary resuscitation (CPR) is often very good.^2–4^ Such technique is obviously reserved to specialized centers, properly equipped and trained in ECMO or CPB placement, with cardiac surgery centers being the main acceptors of such victims. However, the qualification criteria to the procedure, especially in co-existing trauma, can be problematic.

## CASE PRESENTATION

A male patient, no ID, was found in the street of Cracow by the police patrol in early morning. Ambient temperature during that night was 0°C, with 7 to 9 km/h wind and mists. On arrival of ambulance service, the patient was found to be extremely untidy, breathing spontaneously, with palpable carotid pulse, Glasgow Coma Scale (GCS) of 10 and some bruising and contusion on the right temple. Standard prehospital thermal insulation was applied. The patient was transferred to the nearest general hospital. During initial decontamination, the patient started to deteriorate quickly, with severe bradycardia 20 per min and no palpable pulse. CPR according to European Resuscitation Council protocol^5^ with intubation was immediately performed and after 15 min his circulation was restored. After return of spontaneous circulation (ROSC), symptoms of hemodynamic instability occurred (heart rate 50–60 bpm, systolic blood pressure 50–90 mm Hg) and continuous infusions of adrenaline and dopamine in gradually increased doses were needed. The mid-esophageal temperature was 25°C. Standard rewarming techniques were implemented (forced air, warm intravenous fluids, and bladder lavage). Despite those means, symptoms of shock were exacerbating. After consulting the hypothermia coordinator, the patient was subjected to extracorporeal treatment of severe hypothermia protocol.

Due to signs of head contusion and unclear history, a computed tomography (CT) trauma scan was performed that showed a few abnormalities. Head CT showed some small (2–3 mm) pericerebral hematoma over right temporal and parietal lobe with history of previously undergone right craniotomy (no medical notes). There were also fractures of right occipital and parietal bones. Thoraco-abdominal CT showed multiple right-sided rib fractures, 6th thoracic vertebrae fracture, perihepatic and perisplenic fluid with no obvious source of bleeding and abnormal enhancement in pancreas corresponding most likely to acute pancreatitis. According to hypothermia protocol, the patient was a candidate for extracorporeal rewarming (Swiss Staging hypothermia class III/IV), but there was a serious concern regarding heparinization. Therefore, after second discussion with hypothermia coordinator, further investigations were performed. Specialist neurosurgeon on-call opinion was sought, who revised head CT and contributed hemorrhagic findings to past craniotomy. Repeated abdominal ultrasound examinations did not show any dynamic increase in the amount of the intraperitoneal fluid. Acute trauma was ruled out, so after final consultation with hypothermia protocol coordinator, the patient was transferred to John Paul II Hospital for ECMO implantation.

In the Emergency Department, the patient was reassessed—he was unconscious, with wide, unresponsive pupils, intubated, ventilated with low end-tidal carbon dioxide (13 mm Hg). His heart rate was 40 per min (junctional rhythm). The patient was transferred straight to operating theater, where arterial-venous ECMO, through femoral vessels with extraarterial return cannula was surgically implanted. Initial flow was set at 4 L/min. Five thousand units of heparin was used in priming, and further heparin administration was guided by activated clotting time (ACT), to reach the range of 160 to 200 s.

On admission to operating theater the patient was deeply hypotensive with the need of vasoactive drugs support (epinephrine 0.12 μg/kg/hr and norepinephrine 0.12 μg/kg/hr infusions). Acidosis and high lactate levels were found, as well as persistent hypoglycemia (see Table 1). The whole rewarming lasted 87 min, but the ECMO was not terminated, because it served as a respiratory support (the patient had aspirated gastric content during initial CPR) and cardiac support (refractory cardiogenic shock). Persistent hemodynamic instability necessitated bringing the patient back to normothermia. Repeated ultrasound assessment was performed to monitor the incidence of possible hemorrhage due to heparinization and occult trauma.

**TABLE 1:**
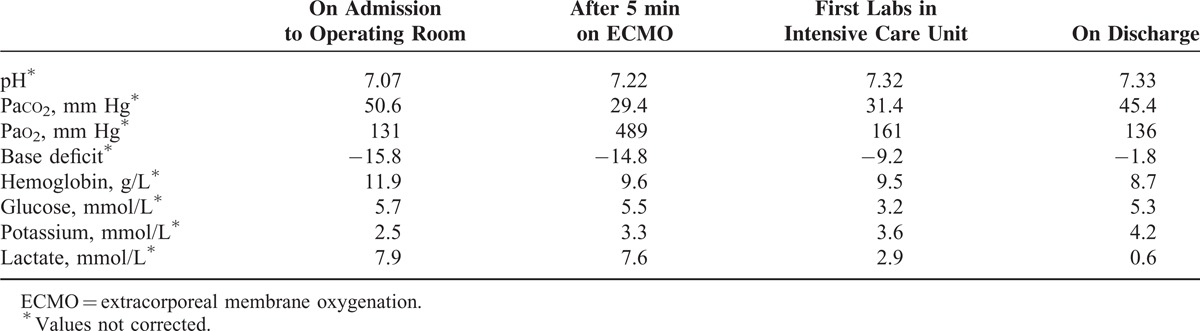
Blood Chemistry Values During Rewarming

Over following 24 h, set cardiac output was gradually reduced and the ECMO was successfully terminated on the next day. The patient remained on conventional mechanical ventilation. Two days later he was examined by the neurologist who found the patient unconscious, with reactive, narrow pupils, flaccid bilateral paralysis and no other pathological findings. On day 5, after repeated head CT, that showed no new findings, the patient was transferred to the intensive care unit in one of the neighboring district hospitals for further treatment of pneumonia (cultures positive for *Escherichia coli*) and general rehabilitation. He was successfully weaned off the ventilator and was extubated on day 8. Neurological re-evaluation showed no obvious disabilities GCS 15; CPC-1. Cardiac function was also preserved with ejection fraction of 50%. In a year follow-up, the patient was found to be alive, with no neurologic deficits.

## DISCUSSION

The case described above appears significant for 2 reasons. Firstly, it was the first such case documented in Poland, that is, of treatment of severe accidental hypothermia using ECMO. Secondly, the case was additionally complex due to co-existing trauma.

Rewarming was possible thanks to development and implementation of a protocol, in which all patients from south-east Poland with confirmed, profound class III and IV hypothermia according to Swiss Staging, are consulted with intensive care unit of the Cardiac Surgery Department in John Paul II Hospital, Cracow, Poland. Basing on inclusion/exclusion criteria, patients are transferred to Severe Accidental Hypothermia Center, where extracorporeal rewarming (ECR) is implemented. For organizational reasons this center is able to accept patients only from south-east Poland, yet, it is a vast land of mainly rural and mountainous countryside (population 3.3 million; area 15,100 km^2^).^6^

Severe hypothermia not complicated by any other medical conditions constitutes an indication for ECR,^5,7^ but co-existent trauma impedes decision making. The basic problem is the necessity of systemic anticoagulation implementation, which may result in hemorrhagic diathesis and serious complications. Each injury, especially of internal organs, may become a source of potentially lethal hemorrhage. Protocols used in various centers postulate abandonment from extracorporeal rewarming in patients with severe blunt or penetrating trauma.^4^ On the other side, some trauma centers proved efficacy and safety of veno-venous ECMO in patients with multiple organ injuries—including those in hypothermia.^8,9^ It should be emphasized that the beneficial effect of ECMO in hypothermia results mainly from 3 mechanisms of action: rewarming, providing cardiovascular support, and maintaining tissue oxygenation. In severe hypothermia with cardiac instability, a prolonged cardiopulmonary support may be essential, and accessible only in venous-arterial configuration and high blood flow.

In the described case, the initial examination indicated existence of clinically important organ trauma. The assessment was complicated by lack of information about the circumstances which led to hypothermia and lack of medical history of the patient. Eventually, after a series of further examinations, consultations with specialists and re-assessment of risks and benefits the decision to implement extracorporeal rewarming was made, despite the co-existing threats. The treatment proved successful and repeated imaging indicated no active bleeding or any other urgent complications.

The difficulties and dilemmas present during the treatment provoked us to pose questions: which trauma should be considered an absolute and which a relative contraindication for ECMO rewarming? What are the limits of use of ECR in hypothermia? While in respiratory failure ECMO is believed to be the “last chance treatment,” in hypothermia a selection of simpler and less invasive accepted alternative methods can be put to use.^10^ Research in available studies brought no clarification of the dilemma. Of those described hypothermic patients rewarmed with ECMO, most had no co-existing trauma. It is possible that clinically insignificant trauma was not mentioned. On the other hand, some of the reports pertained to victims of accidents in the mountains (eg, avalanche victims) or patients after successful resuscitation among whom the likelihood of organ trauma is noticeable.^2^

Our questions remain open. Perhaps development of extracorporeal circulation technique and further experience with hypothermic patients will produce answers. However, the increase in number of patients in accidental hypothermia being treated with extracorporeal techniques will necessitate development of diagnostic and therapeutic criteria for inclusion to ECMO rewarming. In our opinion, repeated imaging (ultrasound and CT) and dynamic assessment of possible occult trauma along with specialist consultation on contraindications for systemic anticoagulation in case of abnormal findings are the keys to balancing risk–benefit ratio in patients with suspected trauma.
